# Supplementary material for: Optimizing PET Glycolysis with an Oyster Shell-Derived Catalyst Using Response Surface Methodology
Source: Polymers (Basel). 2022 Feb 9;14(4):656. doi: 10.3390/polym14040656 (PMC8877978; doi:10.3390/polym14040656)
Supplement: Supplementary file 1 [file polymers-14-00656-s001.zip › polymers-1585634-supplementary.pdf]

Article

Optimizing PET Glycolysis with an Oyster Shell-Derived Catalyst Using Response Surface Methodology

Yonghwan Kim [1,†], Minjun Kim [2,†], Jeongwook Hwang [1], Eunmi Im [3] and Geon Dae Moon [3,*]

[1] Department of Advanced Materials R&D Center, Dae-Il Corporation (DIC), Ulsan 44914, Korea; kyh871111@dicorp.co.kr (Y.K.); jwhwang@dicorp.co.kr (J.H.)
[2] RIKEN Center for Emergent Matter Science (CEMS), Saitama 351-0198, Japan; minjun.kim@riken.jp
[3] Dongnam Division, Korea Institute of Industrial Technology (KITECH), Busan 46938, Korea; eunmi47@kitech.re.kr
* Correspondence: gmoon@kitech.re.kr
† These authors contributed equally to this work.

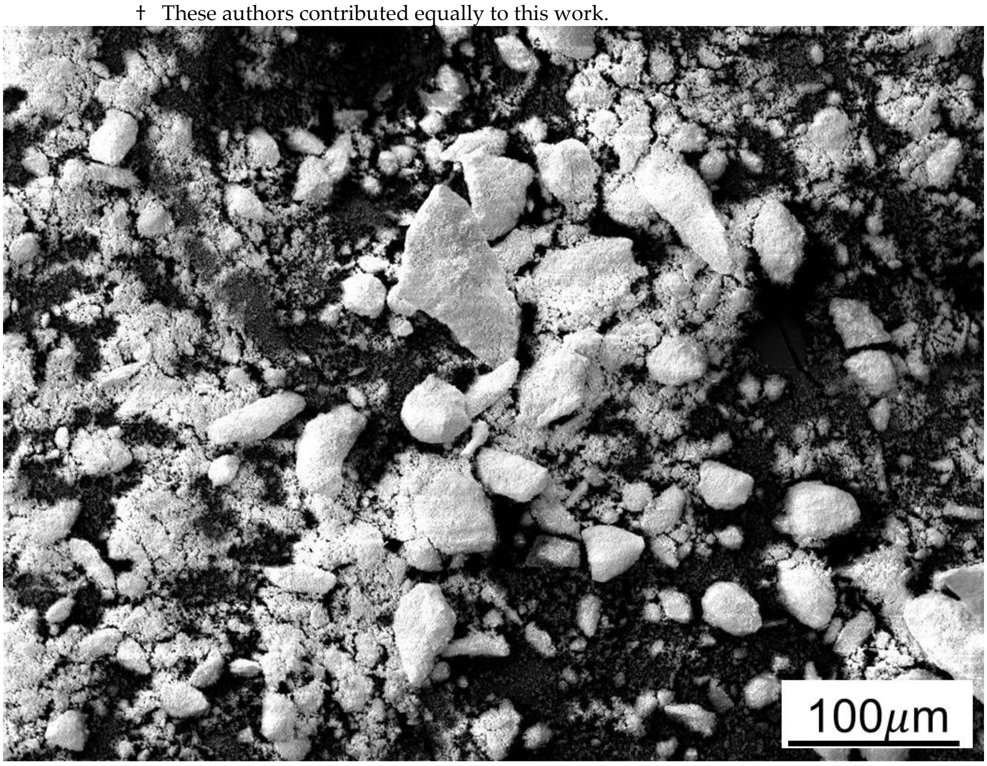

Figure S1. SEM image of uncalcinated oyster shell powder.

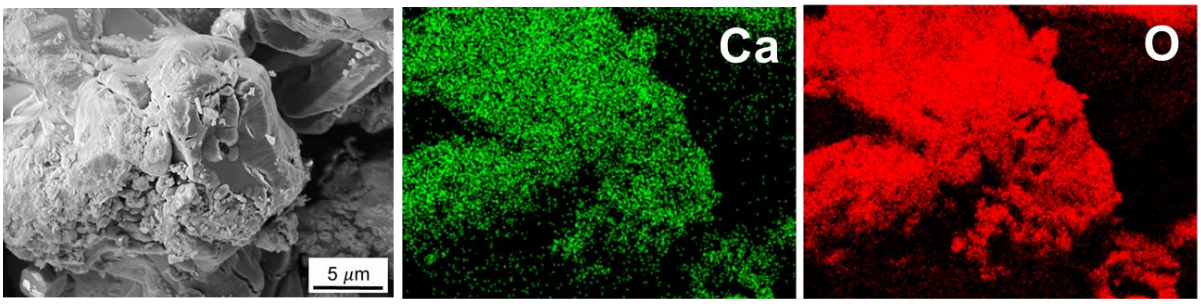

Figure S2. EDS elemental map over a single calcined oystershell particle.

Table S1. EDS results of calcined oyster shells

Element	Wt%
C	2.72
O	25.97
Na	0.25
Mg	0.38
Al	0.26
Si	0.42
Ca	69.99
Total:	100

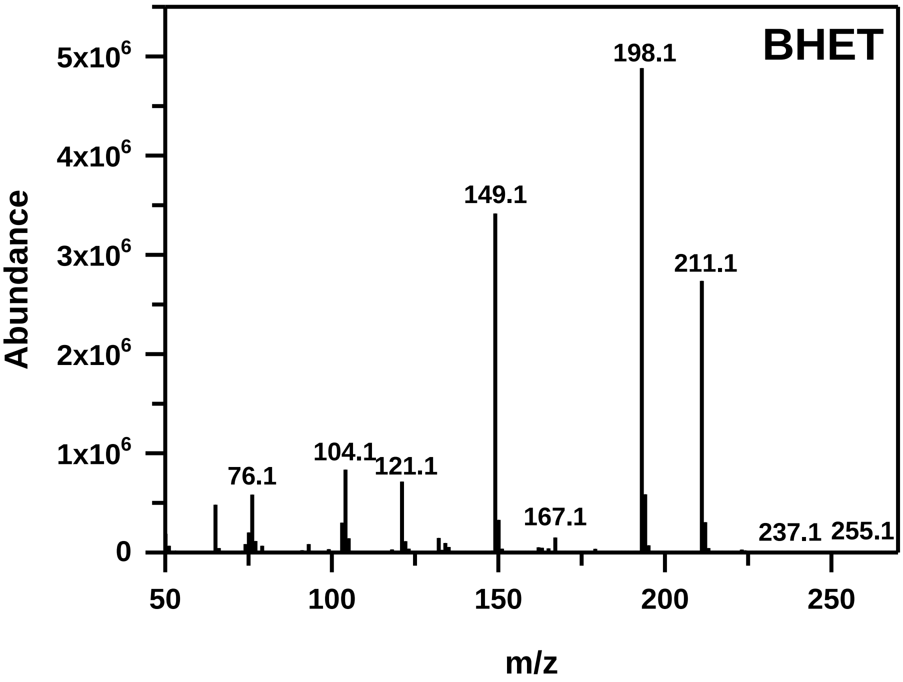

Figure S3. GC-MS spectrum of BHET.

Table S2. Characterization fragments of the glycolysis products in mass spectra.

m/z	Fragments
255	$HO(CH_2)_2OOCC_6H_4COO(CH_2)_2OH$
237	$HO(CH_2)_2OOCC_6H_4COO(CH_2)_2$
211	$HO(CH_2)_2OOCC_6H_4COOH$
193	$HOOCC_6H_4COO(CH_2)_2$
167	$HOOCC_6H_4COOH$
149	$HOOCC_6H_4CO$

132	OCC_6H_4CO
121	$HOOCC_6H_4$
104	C_6H_4CO
76	C_6H_4

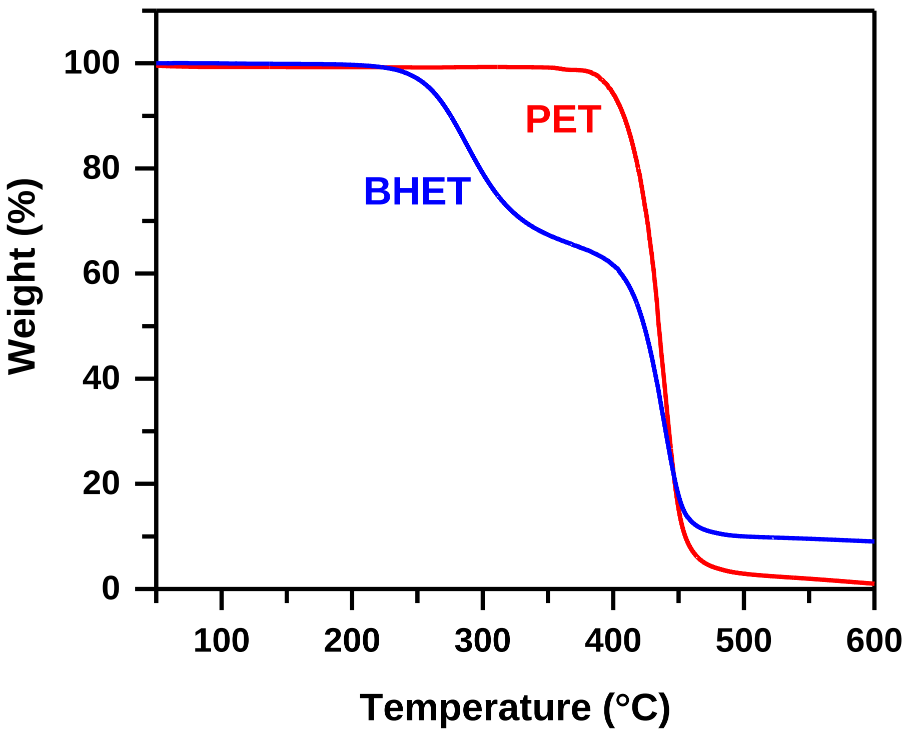

Figure S4. TGA curves of BHET product and PET waste.

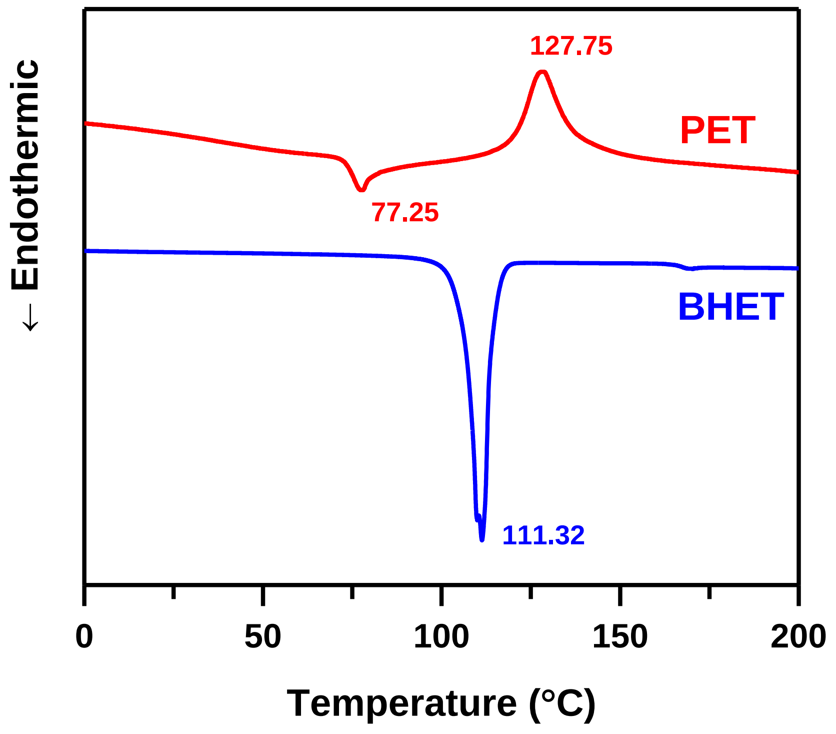

Figure S5. DSC curves of BHET product and PET waste.

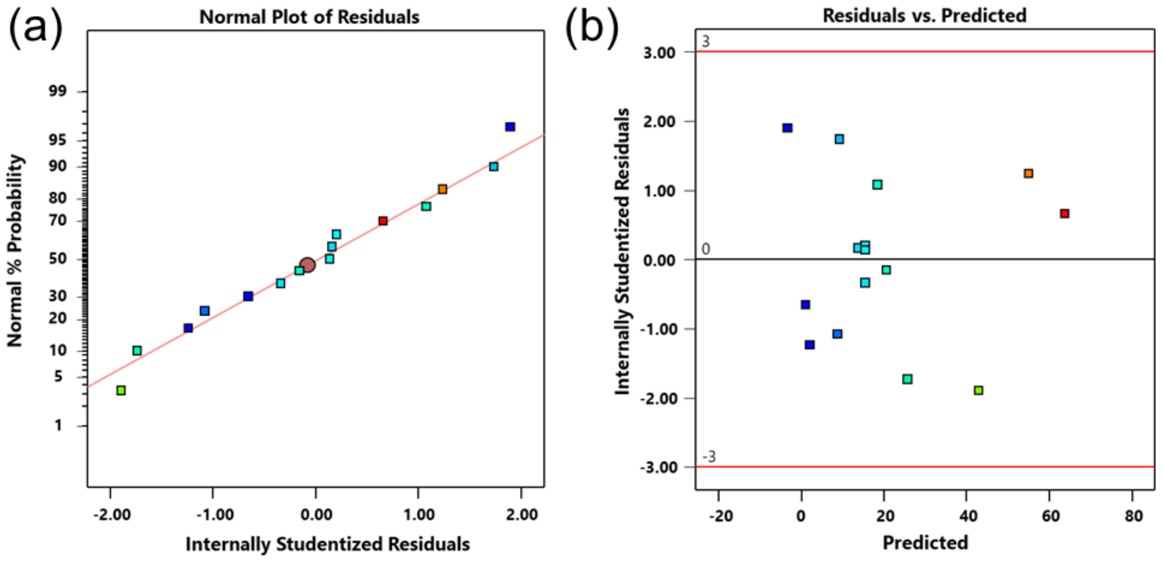

Figure S6. Residual plot of quadratic model: (a) Normal probability plot of the internally studentized residuals, (b) Scatter plot of the internally studentized residuals and predicted value.

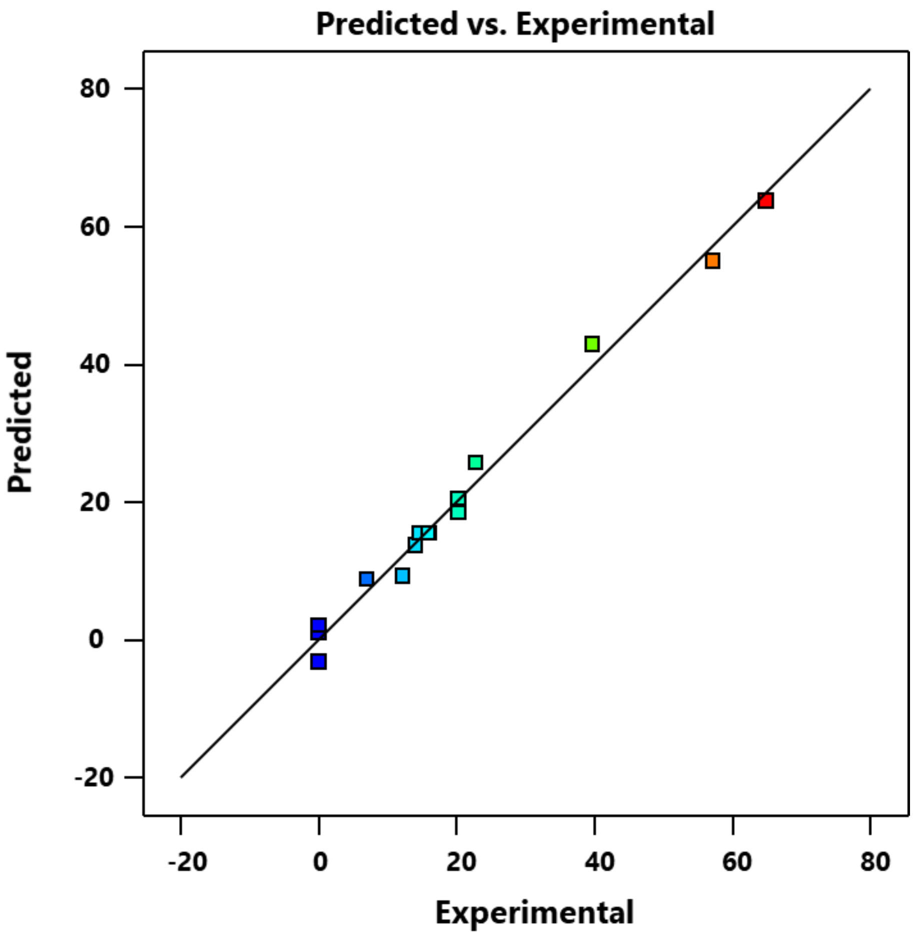

Figure S7. Plot of the actual and predicted value for yield of BHET.
